# The Clinical Effectiveness and Safety of Vaccinations against COVID-19 in HIV-Positive Patients: Data from Observational Study in Poland

**DOI:** 10.3390/vaccines11030514

**Published:** 2023-02-22

**Authors:** Carlo Bieńkowski, Agata Skrzat-Klapaczyńska, Ewa Firląg-Burkacka, Andrzej Horban, Justyna D. Kowalska

**Affiliations:** 1 Hospital for Infectious Diseases in Warsaw, 01-201 Warsaw, Poland; 2 Department of Adults’ Infectious Diseases, Medical University of Warsaw, 01-201 Warsaw, Poland

**Keywords:** HIV, COVID-19, vaccination, prevention, viral infections

## Abstract

People living with HIV (PLWH) are a heterogeneous group of immunocompromised persons, yet underrepresented in randomized clinical trials leading to vaccination registration. Detectable HIV viral load and having chronic comorbidities may increase the risk of severe COVID-19 outcomes in this group of patients. We aimed to assess the efficacy and safety of vaccinations against COVID-19 in PLWH. Materials and Methods: We performed a retrospective analysis of medical records of HIV-positive individuals routinely followed up between 1 January 2021 and 30 April 2022 that were at the HIV Outpatient Clinic in Warsaw. The analysis included data on the type and date of administration of subsequent doses of COVID-19 vaccination, adverse vaccine reactions, and the history of SARS-CoV-2 infection. Results: In total, 217 patients were included in the analysis, with a median age of 43 years (IQR: 35.5–51.5 years) and median CD4+ count of 591 cells/uL (IQR: 459.5–745.0 cells/uL). Most of the patients were male (191/217, 88.0%) and were vaccinated with the BNT162b2 vaccine (143/217, 65.9%). None of the patients diagnosed with COVID-19 required hospitalization. Vaccine adverse events (VAE) mostly occurred after the 1st dose (in 33/217 (15.2%)), and none of them were severe or required medical care. Conclusions: In our cohort of patients, vaccination against COVID-19 proved to be safe and effective against a severe course of the disease among people living with HIV. However, vaccination, to a lesser degree, protects against mild SARS-CoV-2 infection. Longer observations are required in order to assess the sustainability of protection against severe COVID-19 in this group of patients.

## 1. Introduction

In February 2020, the World Health Organization (WHO) declared a new pandemic of the SARS-CoV-2 virus that causes COVID-19. Since then, the race to find a safe and effective vaccine has been a global health priority [1]. The first COVID-19 case in Poland was confirmed on 4th March 2020. Local epidemic waves in Poland have been distinguished according to the observed increase, peak, and decreases in new COVID-19 cases and observed variants of the SARS-CoV-2 virus. According to the observation of a huge cohort of almost 2200 patients that had been under the care of the Hospital for Infectious Diseases in Warsaw in the period from March 2020 to November 2021, the mortality rate varied from 10.7% in the first wave to 16.8% in the third wave [2].

The most vulnerable groups of patients with the highest risk of a severe infection include elderly persons and immunocompromised people. People living with HIV are a non-homogeneous group of immunocompromised persons, which includes both low CD4 count and ongoing immune activation impacting the response differently to and course of other viral infections [3,4]. Detectable viral load and concomitant chronic comorbidities independently increase the risk of severe outcomes of COVID-19 in this group of patients [5,6,7,8].

At the end of December 2020 in Poland, the first vaccine against COVID-19 was introduced (BNT162b2) as part of a national program; however, not everyone was eligible to receive it at the end of 2020 [9]. In the beginning, healthcare workers and elderly persons were prioritized groups, soon followed by immunocompromised patients, including all HIV-positive persons (irrespective of CD4+ lymphocyte count) [2]. Up to April 2021, there were four vaccines available for Polish citizens, including BNT162b2, mRNA-1273, ChAdOx1-nCoV-19, and Ad.26.COV.2-S [10,11,12,13]. However, in the beginning, people living with HIV qualified for vaccination against COVID-19 with the rest of the population in Poland (they were not prioritized), and our study was conducted during that period.

Initially, registrational trials for anti-COVID-19 vaccines did not include people living with HIV. However, after the intervention of activists and scientific societies, including the Polish Scientific AIDS Society, there has been a joint statement recommending the prioritization of this group of patients in being vaccinated against COVID-19 [14]. Countries adapted this call differently; however, Poland introduced it with no restrictions or CD4 count limits [9]. The BNT162b2 vaccine phase 2/3 trial included 196 people living with HIV (0.5% of the study population), but the efficacy and safety data of the vaccine were not reported [15,16]. The mRNA-1273 vaccine trial included 176 people living with HIV (0.6% of all participants), of whom 90 received the vaccine, and none had COVID-19 after 2 doses [16,17]. Nevertheless, there were no separate analyses regarding vaccines’ safety and efficacy in this specific subgroup of patients [15,17]. The only in-depth analyses showing the high immunogenicity of vaccines among people living with HIV were performed for vector vaccines, which, in turn, were not accessible at the beginning of the epidemic, and, if accessible, they were not widely used [18,19].

The aim of our study was, therefore, to assess the efficacy and safety of vaccinations against COVID-19 in a group of people living with HIV routinely followed up after participation in a national vaccination program in Poland.

## 2. Material and Methods

Patients that were routinely followed at the HIV Outpatient Clinic in the Hospital for Infectious Diseases in Warsaw (Poland) were asked about being vaccinated against COVID-19, and the occurrence of adverse events after the procedure. On average, each patient is seen by his HIV specialist each second month. During routine visits, the patients were interviewed about COVID-19 disease history and vaccination status. Each answer provided by the patient was included in medical records, which were later retrospectively analyzed. SARS-CoV-2 infections were symptomatic and self-reported by patients, confirmed either based on typical symptoms and epidemiologic interview (e.g., developing typical symptoms after contacting someone with confirmed COVID-19), real-time polymerase chain reaction assay (RT-PCR) or SARS-CoV-2 antigen test.

The analysis included data on the type and date of administration of subsequent doses of COVID-19 vaccination, self-reported vaccine adverse events (VAE), and the history of SARS-CoV-2 infection. VAEs were defined as any vaccine side effects that were observed up to four weeks after being vaccinated.

Data were collected from the HIV Outpatient Clinic in the Hospital for Infectious Diseases in Warsaw (Poland) records for a period between 1 January 2021 and 30 April 2022. All collected data were anonymized by giving every patient a code and entered into the Excel database. After collecting all the data, they were retrospectively analyzed. In the statistical analysis, non-parametric tests were used as appropriate. All tests were two-sided, and a *p*-value below 0.05 was accepted as significant. All analyses were performed using SAS version 9.4 (SAS Institute, Cary, NC, USA).

### 2.1. Ethical Statement

The study was conducted in accordance with the Declaration of Helsinki and approved by the Medical University of Warsaw’s Bioethical Committee under approval number AKBE/155/2021.

### 2.2. Funding

The study was supported with an unrestricted research grant from the Research Development Foundation at the Hospital for Infectious Diseases in Warsaw.

## 3. Results

Retrospective data for 217 patients consecutively followed up between 1 January 2021 and 30 April 2022 were included in the analysis. In total, 159 patients were vaccinated with the mRNA vaccine (143, 65.5% vaccinated with BNT162b2 and 16, 7.3% with mRNA-1273), 36 (16.5%) with ChAdOx1-nCoV-19, and 22 (10.1%) with Ad.26.COV.2-S (Table 1). Most of the patients were male (191/217, 88.0%) with a median age of 43 years (IQR: 35.5–51.5 years) and a median CD 4+ cell count of 591 (IQR: 459.5–745.0) cells/uL. The HIV viral load was undetectable in 99.5% (216/217) of patients. These characteristics did not vary significantly between the vaccination groups.

Thirty-three patients (15.2%) reported vaccine adverse events (VAE) after the 1st dose of the vaccination, and none of them were severe or required medical care. In addition, the ChAdOx1-nCoV-19 vaccine resulted in more VAEs as compared to BNT162b2, m-RNA-1273, and Ad.26.COV.2-S vaccines: 13/36 (36.1%) vs. 14/143 (9.8%) vs. 2/16 (12.5%), and 4/22 (18.2%), respectively *p* = 0.013 (Table 2).

Eighteen patients (8.3%) were diagnosed with COVID-19 before any vaccination dose, with no significant difference between the vaccination groups (*p* = 0.130). None of the patients diagnosed with COVID-19 required hospitalization or etiotropic treatment.

The effectiveness, measured as the incidence of symptomatic SARS-CoV-2 infection during the study period compared after a complete primary series of vaccination, was nine (5.7%) for mRNA vaccines, two (5.6%) for ChAdOx1-nCoV-19, and three (13.6%) for Ad.26.COV.2-S (*p* = 0.351). There were 12 (7.5%) SARS-CoV-2 infections reported after a boosted dose in the mRNA vaccines group and none after the booster in ChAdOx1-nCoV-19 and Ad.26.COV.2-S groups (*p* = 0.136), Table 2.

## 4. Discussion

In Central and Eastern Europe (CEE), strategies for vaccination against COVID-19 have varied depending on the country. Jilich D. et al., as part of the Euroguidelines in the Central and Eastern Europe Network Group study, analyzed national strategies for vaccination against COVID-19 among people living with HIV in CEE. The analysis revealed that only 8/21 (38%) countries (the Czech Republic, Greece, Hungary, Lithuania, Montenegro, Romania, Slovakia, and Slovenia) included people living with HIV in the priority group for vaccination, and only 3 (14.2%) countries (the Czech Republic, Greece, Serbia) had national guidelines for the vaccination of people living with HIV. Some countries prioritized all HIV-positive persons (e.g., the Czech Republic, Poland), while others prioritized only those people with advanced HIV disease (e.g., Slovakia) [9].

On the other hand, there is still another issue worth mentioning, which is the willingness to be vaccinated. Babicki et al. performed a cross-sectional survey study in order to see the attitudes toward vaccination against COVID-19 in the Polish population. They performed the same questionnaire before and two months after the commencement of the National Vaccination Programme in Poland. They showed that women and elderly persons (>60 years of age) living in cities >250,000 inhabitants, with higher education, married, healthcare professionals, and people that were previously vaccinated were more likely to have a positive attitude towards vaccination against COVID-19. In addition, people with chronic diseases did not show any significant difference in this regard compared to the rest of the study participants. However, in this study, people living with HIV were not distinguished as a separate group of persons with chronic diseases but were probably included in the subgroup of “other”. Moreover, convalescents did not seem to be more likely to be vaccinated against COVID-19 [20]. Kaida et al. performed a survey on people living with HIV and their intention to receive the vaccine against COVID-19 and showed that intention to vaccinate was significantly lower among people living with HIV compared to participants not living with HIV (65.2% vs. 79.6%; OR 0.44; 95% CI 0.32–0.60). However, this association was not statistically significant after adjusting for ethnicity, income, education, and essential worker status (aOR 0.85; 95% CI 0.48–1.55) [21]. Moreover, Su et al. performed a similar survey, but only within the HIV-positive participants, and concluded that the most important factors influencing acceptance were the perception that vaccination is unsafe for HIV-infected people (aOR  =  0.082, 95% CI  =  0.024–0.282) and the poor efficacy in preventing COVID-19 in HIV-positive persons (aOR  =  0.093, 95% CI  =  0.030–0.287). Other factors associated with acceptance included Zhuang ethnicity (aOR  =  1.653, 95% CI  =  1.109–2.465), a highest education level of middle school, high school, or above (aOR  =  1.747, 95% CI  =  1.170–2.608; aOR  =  2.492, 95% CI  =  1.326–4.682), and the vaccination having little effect on ART efficacy (aOR  =  2.889, 95% CI  =  1.378–6.059) [22]. Therefore, there are many issues that clinicians have to face in order to obtain immunity among the vast majority of the population and break the epidemiological chain of SARS-CoV-2 infections. In addition, it is worth mentioning that COVID-19 convalescents are crucial participants in the national vaccination program, especially given that, in this group, a higher immunological response after vaccination is observed [23]. Therefore, it is very important to encourage people living with HIV who have not had COVID-19 and those who are COVID-19 convalescents to complete the vaccination schedules.

The COVID-19 vaccines’ registrational studies showed that these vaccines are safe and have an efficacy of 95%, 94.1%, 62.1%, and 66.9%, respectively, for BNT162b2, mRNA-1273, ChAdOx1-nCoV-19, and Ad.26.COV.2-S against COVID-19 [15,17,24,25]. Our results from real-world vaccine implementation among people living with HIV also confirmed high efficacy across all four vaccines registered in the European Union. These results are in line with our earlier findings that people living with HIV who are on effective combined antiretroviral treatment (cART) have a high immunological response after both the 1st and 2nd vaccine doses of mRNA vaccines [26].

Coburn et al. analyzed the post-vaccination breakthrough SARS-CoV-2 infections among adults with HIV in the United States and compared them to adults without HIV. In total, they had a cohort of 113,994 participants and showed a cumulative incidence of COVID-19 of 3.8% (95% CI, 3.7–3.9%) nine months after full vaccination, although this number was higher in people living with HIV (4.4% vs. 3.5%; log-rank *p* < 0.001). The breakthrough infection risk was also higher in people living with HIV (28%, adjusted hazard ratio, 1.28 (95% CI 1.19–1.37)). In addition, among people living with HIV, a younger age (<45 years vs. 45–54 years), a history of COVID-19, and not receiving an additional vaccine dose (aHR, 0.71 (95% CI, 0.58–0.88)) were associated with an increased risk of breakthrough SARS-CoV-2 infections. Moreover, there was no association with HIV viral load suppression, but a high CD4+ count (i.e., ≥500 cells/mm^3^) was associated with fewer breakthroughs among people living with HIV [27].

However, Hoffman et al., in a multivariate analysis of a cohort of 175 people living with HIV, showed that the only factor associated with risk for severe COVID-19 was a current CD4+ T cell count of <350/µL (adjusted odds ratio 2.85, 95% confidence interval 1.26–6.44, *p* = 0.01) [28]. In our cohort, the median CD4+ count was 591 cells/uL (IQR: 459.5–745.0 cells/uL). Therefore, our cohort had fewer breakthroughs (11/217, 5.1%) after full vaccination (at least two doses in a two-dose regimen), and none of them were severe or required medical care. Nevertheless, as shown in our study, 5% of patients after primary vaccination and 5% after the booster dose acquired SARS-CoV-2 infection. This underlines the importance of continuing other epidemic control, such as wearing masks in public spaces, especially hospitals and transportation hubs [29].

According to the systematic review by Graña et al., the reduction of the incidence of symptomatic COVID-19 while compared to placebo ranged as follows—for BNT162b2: 97.84%, 95% CI 44.25% to 99.92% (2 RCTs, 44,077 participants); mRNA-1273: 93.20%, 95% CI 91.06% to 94.83% (2 RCTs, 31,632 participants); ChAdOx1: 70.23%, 95% CI 62.10% to 76.62% (2 RCTs, 43,390 participants); and Ad26.COV2.S: 66.90%, 95% CI 59.10% to 73.40% (1 RCT, 39,058 participants). In addition, serious VAEs after mRNA-1273, ChAdOx1, and Ad26.COV2.S probably demonstrate little or no difference compared to placebo (RR: mRNA-1273: 0.92, 95% CI 0.78 to 1.08 (2 RCTs, 34,072 participants); ChAdOx1: 0.88, 95% CI 0.72 to 1.07 (7 RCTs, 58,182 participants); Ad26.COV2.S: 0.92, 95% CI 0.69 to 1.22 (1 RCT, 43,783 participants); evidence for severe VAE after BNT162b2 is uncertain compared to placebo (RR: BNT162b2: 1.30, 95% CI 0.55 to 3.07; 2 RCTs, 46,107 participants) [30]. While some of these studies allowed the recruitment of people living with HIV, none of them presented separate analyses for this subgroup of patients.

In clinical practice, all vaccinations available in Poland proved to be safe, with only 15% of patients reporting any VAE, and all of these were mild and self-relieving with time. However, and in parallel to other research, we found ChAdOx1-nCoV-19 to cause more VAEs [18,19,24].

## 5. Limitations

This is an observational study of retrospective character without a control group with an observation period of four months. The study group consists mostly of people living with HIV with undetectable VL and a CD4 count >500 cells/uL. Therefore, we could not assess the vaccines’ safety and efficacy among people living with HIV who are immunocompromised. In addition, a randomized control trial in a group of people living with HIV is required in order to provide a deeper view of the vaccines’ safety and efficacy in this group. However, there are some strengths worth mentioning, including a large sample for such a short period of time and a subgroup of patients who were vaccinated with four different vaccines, which gives us some insight into a deeper understanding of the vaccines’ safety and efficacy in people living with HIV in Poland.

## 6. Conclusions

Vaccination against COVID-19 is safe and effective against a severe course of the disease among people living with HIV. However, longer observation is required to measure the waning of vaccine effectiveness against severe and moderate COVID-19 in this group of patients. Adequate recruitment of people living with HIV to randomized clinical trials of COVID-19 vaccinations, along with presenting results separately for this group of patients, remains crucial to understanding these effects.

## Figures and Tables

**Table 1:** Baseline characteristics of the HIV-positive individuals vaccinated against COVID-19 stratified by the type of first vaccination dose. Presented as number and column percent.

Characteristic	All	BNT162b2	mRNA-1273	mRNA	ChAdOx1-nCoV-19	Ad.26.COV.2-S	*p*-Value *
**All**	217 (100.0)	143 (100.0)	16 (100.0)	159 (100.0)	36 (100.0)	22 (100.0)	-
**Baseline characteristics**							
**Women**	27 (12.4)	21 (14.7)	2 (12.5)	23 (14.5)	2 (5.5)	2 (9.1)	0.484 **
**Age < 60 years**	196 (90.3)	128 (89.5)	13 (81.3)	141 (88.7)	33 (91.7)	22 (100.0)	0.011
**HIV VL < 50 copies/mL**	216 (99.5)	142 (99.3)	16 (100.0)	158 (99.4)	35 (97.2)	22 (100.0)	0.464 ***
**CD4+ < 200 cells/uL**	6 (2.8)	5 (3.5)	0 (0.0)	5 (3.1)	0 (0.0)	0 (0.0)	0.759 **
**CD4+ < 350 cells/uL**	24 (11.1)	16 (11.2)	1 (6.2)	17 (10.7)	5 (13.9)	1 (4.5)	0.652 ***
**CD4+ < 500 cells/uL**	75 (34.6)	51 (35.7)	6 (37.5)	57 (35.8)	11 (30.5)	6 (27.3)	0.826 ***
**COVID-19 before vaccination**	18 (8.3)	12 (8.4)	2 (12.5)	14 (8.8)	0 (0.0)	2 (9.1)	0.130 **

* *p*-values were calculated for mRNA, ChAdOx1-nCoV-19 and Ad.26.COV.2-S comparison. ** Freeman–Halton extension of Fisher’s exact probability test for a two-row by three-column contingency table. *** *p*-values were calculated for BNT162b2, mRNA-1273, ChAdOx1-nCoV-19, and Ad.26.COV.2-S comparison. HIV VL—human immunodeficiency virus viral load.

**Table 2:** Safety and rate of symptomatic SARS-CoV-2 infection data for HIV-positive individuals vaccinated against COVID-19 stratified by the type of first vaccination dose. Presented as number and column percent.

Characteristic	All	BNT162b2	mRNA-1273	mRNA	ChAdOx1-nCoV-19	Ad.26.COV.2-S	*p*-Value *
**All**	217 (100.0)	143 (100.0)	16 (100.0)	159 (100.0)	36 (100.0)	22 (100.0)	-
**Vaccine safety**							
**VAE after 1st vaccine dose**	33 (15.2)	14 (9.8)	2 (12.5)	16 (10.1)	13 (36.1)	4 (18.2)	0.0013 ***
**Rate of symptomatic SARS-CoV-2 infection**							
**After the 1st dose**	4 (1.8)	0 (0.0)	0 (0.0)	0 (0.0)	1 (2.8)	N/A	0.185 **
**After the primary vaccination**	11 (5.1)	9 (6.3)	0 (0.0)	9 (5.7)	2 (5.6)	3 (13.6)	0.351 ***
**After boosted dose**	12 (5.5)	12 (8.4)	0 (0.0)	12 (7.5)	0 (0.0)	0 (0.0)	0.136 **

* *p*-values were calculated for mRNA, ChAdOx1-nCoV-19, and Ad.26.COV.2-S comparison. ** Freeman–Halton extension of Fisher’s exact probability test for a two-row by three-column contingency table. *** *p*-values were calculated for BNT162b2, mRNA-1273, ChAdOx1-nCoV-19, and Ad.26.COV.2-S comparison.

## Data Availability

The data sets used and/or analyzed during the current study can be made available by the corresponding author upon reasonable request. The data are not publicly available due to ethical concerns.
